# Real-world evidence for atropine titration in myopia control: a comparative study of three low-dose regimens in Chinese children

**DOI:** 10.3389/fphar.2026.1716698

**Published:** 2026-03-03

**Authors:** Hui-Xia Li, Peng Wu, Wen-Ping Qi, Li-Li Yang, Hong-Mei Jiang, Dong-Sheng Liang, Gang Bai, Jian-Hua Wu, Li-Xin Song, Xiao-Ying Wu, Jian Liu, Han Zhang, Caihan Qiqige, Gui-Sen Zhang

**Affiliations:** 1 Department of refractive surgery, Inner Mongolia Chaoju Eye Hospital, Hohhot, China; 2 Department of refractive surgery, Baotou Chaoju Eye Hospital, Baotou, China; 3 Department of refractive surgery, Chifeng Chaoju Eye Hospital, Hohhot, China; 4 Department of refractive surgery, Hohhot Chaoju Eye Hospital, Hohhot, China

**Keywords:** axial length, low-dose atropine, myopia control, pediatric ophthalmology, spherical equivalent

## Abstract

**Purpose:**

To evaluate and compare the efficacy of 0.01%, 0.025%, and 0.05% atropine eye drops in slowing myopia progression among Chinese children aged 6–18 years, focusing on changes in spherical equivalent (SE) and axial length (AL).

**Methods:**

In this prospective, real world based, multi-center study, 175 children with myopia were followed for 12 months at three tertiary ophthalmic hospitals in China. Participants received nightly instillations of 0.01%, 0.025%, or 0.05% atropine in affected eyes. Primary outcomes included changes in SE and AL. Secondary outcomes involved corneal curvature, intraocular pressure, and biometric parameters. Statistical analyses included repeated-measures ANOVA, linear regression, and seemingly unrelated regression models.

**Results:**

All three concentrations demonstrated effectiveness in reducing myopia progression. At 12 months, SE progression was lowest in the 0.05% group (−0.27 ± 0.72 D), followed by 0.025% (−0.35 ± 0.59 D) and 0.01% (−0.44 ± 1.02 D), with a significant difference between 0.05% and 0.01% (P = 0.014). AL elongation was numerically lowest in the 0.025% group (0.21 ± 0.19 mm), while differences among groups were not statistically significant (P = 0.299). Regression analysis showed that AL change explained over 34% of SE variation in the 0.025% and 0.05% groups, compared to 14% in the 0.01% group.

**Conclusion:**

Low-concentration atropine is effective in controlling myopia progression in children. Among the three concentrations, 0.025% atropine offers better efficacy and tolerability, providing comparable axial elongation control to 0.05% with potentially fewer side effects. These findings support its use as a first-line pharmacologic option for pediatric myopia management in clinical practice.

## Introduction

Myopia has become a major global public health concern, particularly in East Asia, where prevalence among school-aged children continues to rise at an alarming rate (Holden et al., 2016). In China, over 50% of children aged 6 to 18 are affected by myopia, with a substantial proportion progressing to high myopia during adolescence (Wei et al., 2024). The prevalence of high myopia among adolescents aged 16 to 18 is projected to increase significantly, rising from 7.3% in 2001 to an estimated 22.1% by the year 2050 in China (Pan et al., 2025).

While various optical and behavioral interventions have been explored for myopia control, pharmacologic approaches have gained increasing attention (Xu and Zhang, 2025). Atropine, a non-selective muscarinic antagonist, has shown promising efficacy in slowing myopia progression. The Atropine for the Treatment of Myopia (ATOM) studies (Chia et al., 2016; Hieda et al., 2021) and the Low-Concentration Atropine for Myopia Progression (LAMP) study (Yam et al., 2019) provided robust clinical evidence supporting the use of low-concentration atropine eye drops.

In the ATOM2 study, 0.01% atropine showed a mean 2-year myopia progression of −0.49 D and axial elongation of 0.41 mm, compared to less progression in higher concentrations. Notably, refractive error progression in the 0.01% group slowed significantly in the second year, but axial elongation remained substantial, indicating its limited effect on axial length growth (Chia et al., 2012). However, ATOM2 study lacking a placebo-controlled group limited the strength of its conclusions. The subsequent LAMP study, a double-blinded, randomized, placebo-controlled trial, evaluated 0.05%, 0.025%, and 0.01% atropine and revealed a clear concentration-dependent effect, with 0.05% being the most effective in reducing both spherical equivalent progression and axial elongation, while maintaining good safety and tolerability (Yam et al., 2019).

Despite compelling evidence from multi-ethnic trials, data specific to Chinese pediatric populations under real-world conditions remain limited. Furthermore, the relative effectiveness and safety profile of these concentrations across diverse clinical settings require further validation.

The objective of this prospective study is to evaluate and compare the efficacy of 0.05%, 0.025%, and 0.01% atropine in controlling myopia progression, specifically spherical equivalent (SE) and axial length (AL) changes, among Chinese children aged 6–18 years. This study also aims to provide further insight into the dose-response relationship of low-concentration atropine and inform optimized treatment regimens for clinical practice.

## Methods

### Study design and setting

This was a prospective, real-world, non-randomized, quasi-experimental multicenter study conducted in three tertiary eye hospitals in China including Inner Mongolian Chaoju Eye Hospital (Hohhot), Chifeng Chaoju Eye Hospital (Chifeng), and Baotou Chaoju Eye Hospital (Baotou) from 20 June 2024 to 10 August 2025. The study enrolled children with myopia who met the eligibility criteria and were followed for at least 12 months. The study adhered to the principles outlined in the Declaration of Helsinki and was approved by the Ethics Committee of Inner Mongolian Chaoju Eye Hospital (No. CJYKLLSC20230301). Additionally, written informed consent was obtained from at least one parent or legal guardian of each participant. The trial was prospectively registered with the Chinese Clinical Trial Registry (ChiCTR) under registration number ChiCTR2400085795.

### Sample size

Sample size was calculated using the formula $n=\frac{{\left(Z_{\alpha /2}+Z_{\beta }\right)}^{2}\times \sigma^{2}}{\delta^{2}}$ , where σ represents the standard deviation of annual SE progression, δ represents the minimum clinically meaningful difference, $Z_{\alpha /2}=1.96$ for a two-sided α of 0.05, and $Z_{\beta }=1.28$ for 90% power. Additionally, Sample size estimation was based on data from previous studies (Clark and Clark, 2015; Wei et al., 2020). We assumed that 0.01% atropine would reduce myopia progression by at least −0.36 D over 1 year, with a standard deviation of 0.70 D, using a two-sided significance level of 5% and a statistical power of 90%. Based on these parameters, a minimum of 60 participants per group was required. Accounting for an estimated dropout rate of 20%, a total enrollment of approximately 180 participants was planned. Sample size estimation was performed for the overall study population rather than per center, as data from all participating centers were pooled for analysis under a unified protocol.

### Participants

Children aged 6–18 years with SE refractive error between −1.00 D and −6.00 D and astigmatism of −1.50 D or less in both eyes were eligible. The exclusion criteria included ocular pathology including strabismus, amblyopia, retinal disease, systemic diseases, prior use of atropine or other myopia control treatments (pirenzepine, contact lenses, bifocals, or progressive addition lenses for myopia), or any history of ocular surgery, or best-corrected visual acuity (BCVA) worse than 0.0 logMAR in affected eye. Participants were consecutively recruited from outpatient clinics at participating centers.

Participants received one of three concentrations of atropine eye drops, 0.05%, 0.025%, or 0.01%, administered once nightly in both eyes, based on physician recommendation. All eyedrops in monodose preparation were prepared by Baotou Chaoju Eye Hospital. Participants were advised to avoid concurrent use of other myopia control methods including orthokeratology, multifocal soft contact lenses, progressive addition lenses, bifocal spectacles, or pharmacologic agents such as pirenzepine throughout the study period.

### Data collection and ocular examination

Baseline demographic and ocular biometric data were collected for all participants at enrollment. Demographic data included age, and sex. Ocular assessments included measurements of SE (in diopters, D), AL (mm), average corneal curvature (D), flattest meridian (K1), steepest meridian (K2), white-to-white corneal diameter (WTW, mm), central corneal thickness (CCT, in μm), and intraocular pressure (IOP, mmHg). AL, keratometry, WTW, and CCT were measured using optical biometry (IOL Master, Carl Zeiss Meditec AG, Germany), and IOP was assessed with a non-contact tonometer (Topcon CT-800, Topcon Corporation, Japan). WTW also referred to as horizontal visible iris diameter. Each IOP measurement was obtained as the mean of three consecutive readings. Cycloplegia was achieved using two drops of 1% cyclopentolate administered 5 min apart, with refraction performed 30 min after the last instillation. Three consecutive autorefraction readings were obtained and averaged. Cycloplegic autorefraction was selected due to its high reproducibility and feasibility in large pediatric cohorts. All measurements were conducted by trained technicians following standardized protocols to ensure consistency across study sites.

### Outcomes

The SE was calculated as the dioptric powers of the sphere and half of the cylinder (sphere +1/2 × cylinder). Secondary outcomes comprised corneal curvature (K1, K2, average K), incidence of adverse effects such as photophobia, near vision blur, and self-reported compliance.

### Follow-up schedule

Primary outcomes were defined as 12-month changes; secondary analyses evaluated changes at interim follow-up visits (1, 3, 6, and 9 months).

### Statistical analysis

All statistical analyses were performed using R software (version 4.5.0; R Foundation for Statistical Computing, Vienna, Austria). Continuous variables included SE, AL, keratometry values, WTW, CCT, and IOP; categorical variables included sex and incidence of adverse events. Continuous variables are presented as mean ± standard deviation (SD), and categorical variables as frequencies and percentages. Repeated-measures analysis of variance (ANOVA) was used to evaluate changes in SE and AL over time between treatment groups. Other continuous secondary outcomes were analyzed using one-way ANOVA or the Kruskal–Wallis test, depending on data distribution, with Bonferroni correction applied for *post hoc* comparisons. Categorical variables were compared using the chi-square test or Fisher’s exact test, as appropriate. To examine associations between treatment and continuous outcomes, linear regression analyses were conducted using two modeling strategies. Model 1 was constructed using a stepwise selection procedure to identify significant covariates, whereas Model 2 was fitted using the enter method, in which all prespecified covariates were simultaneously included. Regression coefficients (β) with corresponding standard errors and 95% confidence intervals (CIs) were reported. Adjusted *R* ^2^ values were calculated to assess the proportion of variance explained by the independent variables in each model. A two-sided P value <0.05 was considered statistically significant.

## Results

Initially, a total of 225 children were initially screened for eligibility. Of these, 18 did not meet the inclusion criteria, 11 met the exclusion criteria, and 6 declined participation. As a result, 190 children were enrolled and assigned to one of the three treatment groups (0.05%, 0.025%, or 0.01% atropine) based on parental preference and clinical recommendation. During the 12-month follow-up, 15 participants were lost to follow-up or withdrew from the study. Ultimately, a total of 175 children, 59 in the 0.05% group, 60 in the 0.025% group, and 56 in the 0.01% group completed the study and were included in the final analysis. Baseline characteristics are shown in Table 1. There was a statistically significant difference in age among the groups (P = 0.004), with children in the 0.05% atropine group being slightly younger on average. No significant differences were observed in sex distribution, baseline SE, AL, average keratometry (K), K1, K2, WTW, CCT, or IOP (all P > 0.05). There were also no significant interocular differences except for CCT (P = 0.005), supporting the reliability of monocular analysis. Therefore, only the right eye was included in the analysis.

**TABLE 1:** Baseline demographics and ocular parameters of study participant.

​	Completed 1 Year follow-up (n = 175)			​
	0.05% atropine group (n = 59)	0.025% atropine group (n = 60)	0.01% atropine group (n = 56)	P^b^ value
Age (years)	9.25 ± 1.95	10.23 ± 1.70	10.34 ± 1.59	**0.004**
Male gender, no. (%)	28 (47.5%)	31 (51.7%)	28 (50%)	0.899
Spherical equivalent (D)				
OD	−1.80 ± 1.56	−2.04 ± 1.06	−2.08 ± 1.69	0.105
OS	−1.73 ± 1.75	−1.95 ± 1.25	−2.08 ± 1.56	0.190
P^a^ value	0.354			​
Axial length (mm)				
OD	23.99 ± 0.91	24.15 ± 0.80	24.17 ± 0.79	0.440
OS	23.89 ± 0.87	24.17 ± 0.91	24.17 ± 0.77	0.131
P^a^ value	0.118			​
Average corneal curvature (D)				
OD	42.84 ± 5.93	43.59 ± 1.40	43.59 ± 1.39	0.967
OS	43.71 ± 1.16	43.59 ± 1.48	43.60 ± 1.45	0.631
P^a^ value	0.969			​
Flattes (K1)				
OD	42.19 ± 5.84	43.00 ± 1.34	42.82 ± 1.37	0.763
OS	43.02 ± 1.24	42.98 ± 1.41	42.80 ± 1.45	0.445
P^a^ value	0.189			​
Steepest (K2)				
OD	43.49 ± 6.03	44.18 ± 1.51	44.37 ± 1.56	0.832
OS	44.39 ± 1.16	44.21 ± 1.60	44.40 ± 1.58	0.720
P^a^ value	0.270			​
WTW (mm)				
OD	11.87 ± 1.65	12.02 ± 0.39	12.07 ± 0.32	0.752
OS	11.90 ± 1.67	12.02 ± 0.50	12.05 ± 0.42	0.851
P^a^ value	0.416			​
CCT (mm)				
OD	533.02 ± 78.41	544.73 ± 34.18	537.89 ± 36.59	0.656
OS	531.35 ± 77.87	542.03 ± 33.98	536.79 ± 37.90	0.840
P^a^ value	**0.005**			​
IOP (mmHg)				
OD	16.42 ± 2.80	16.39 ± 3.70	16.82 ± 3.01	0.706
OS	16.53 ± 2.58	16.98 ± 4.08	16.75 ± 2.75	0.410
P^a^ value	0.097			​

P^a^ values were derived from Wilcoxon signed-rank tests for paired interocular comparisons; P^b^ values reflect intergroup comparisons (ANOVA, for normally distributed variables/Kruskal–Wallis for nonparametric data); CCT, central corneal thickness; D = diopter; IOP , intraocular pressure. WTW: White-to-White distance; Normally distributed data: mean ± SD; Non-normally distributed data: median (IQR); P < 0.05 was considered statistically significant. The bold values mean statistical significant.

**FIGURE 1:**
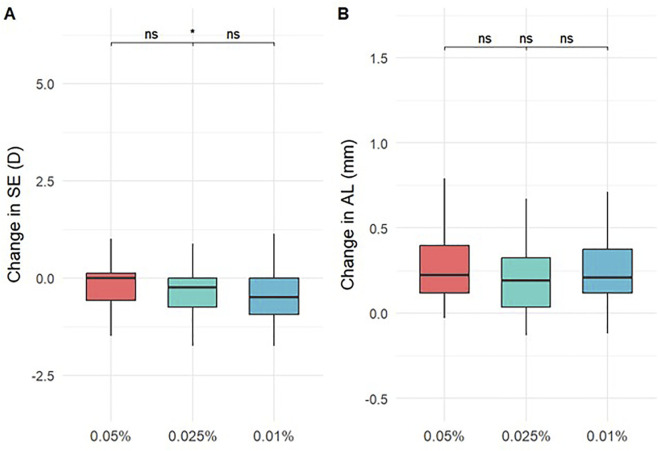
Changes in spherical equivalent (SE) **(A)** and axial length (AL) **(B)** over 12 months.

Changes in SE and AL over 12 months are summarized in Table 2 and Figure 1. The mean change in SE was −0.27 ± 0.72 D in the 0.05% group, which was significantly less than that in the 0.01% group (−0.44 ± 1.02 D, P = 0.014), although the difference compared to the 0.025% group (−0.35 ± 0.59 D) did not reach significance (P = 0.279). Axial elongation was numerically lowest in the 0.025% group (0.21 ± 0.19 mm), followed by the 0.05% group (0.23 ± 0.22 mm) and 0.01% group (0.27 ± 0.23 mm). However, group differences in AL change were not statistically significant (P = 0.299). Group differences in SE progression were already evident at 1, 3, and 6 months (P < 0.05), particularly between the 0.05% and 0.01% groups, while AL changes showed no significant differences at any time point.

**TABLE 2:** Changes in refractive error and ocular biometrics in 0.05% atropine, 0.025% atropine, and 0.01% atropine groups at each visit.

​	Mean ± SD			Overall P value	P value (in pair Comparisons)		
Change between	0.05% atropine group (n = 59)	0.025% atropine group (n = 60)	0.01% atropine group (n = 56)		0.05% atropine vs. 0.025% Atropine	0.05% atropine vs. 0.01% Atropine	0.025% atropine vs. 0.01% Atropine
Baseline and 1 mos							
Δ spherical equivalent (D)	0.08 ± 0.40	−0.15 ± 1.49	0.10 ± 0.31	**0.002**	**0.003**	1.000	**0.005**
Δ axial length (mm)	0.02 ± 0.12	0.02 ± 0.08	0.01 ± 0.04	0.976	1.000	1.000	1.000
Δ average K (D)	0.06 ± 8.33	−0.03 ± 0.34	0.02 ± 0.19	0.789	0.795	0.868	1.000
Δ flattest K (D)	0.86 ± 5.71	−0.05 ± 0.39	0.03 ± 0.37	0.284	0.342	0.202	1.000
Δ steepest K (D)	0.84 ± 6.01	−0.01 ± 0.38	0.00 ± 0.21	0.599	0.482	0.747	1.000
Baseline and 3 mos							
Δ spherical equivalent (D)	0.05 ± 0.48	−0.09 ± 0.34	−0.00 ± 0.38	**0.040**	**0.018**	0.163	0.533
Δ axial length (mm)	0.06 ± 0.14	0.05 ± 0.10	0.07 ± 0.17	0.499	0.884	0.787	0.358
Δ average K (D)	0.83 ± 6.03	0.01 ± 0.23	−0.03 ± 0.40	0.687	1.000	0.896	0.587
Δ flattest K (D)	0.80 ± 5.88	−0.03 ± 0.22	0.01 ± 0.57	0.693	0.587	1.000	0.981
Δ steepest K (D)	0.86 ± 6.20	0.05 ± 0.31	−0.07 ± 0.48	0.147	1.000	0.177	0.109
Baseline and 6 mos							
Δ spherical equivalent (D)	0.01 ± 0.56	−0.15 ± 0.56	−0.21 ± 0.46	**0.004**	**0.008**	**0.004**	1.000
Δ axial length (mm)	0.13 ± 0.16	0.11 ± 0.14	0.13 ± 0.20	0.730	0.653	1.000	0.925
Δ average K (D)	0.86 ± 5.98	−0.28 ± 2.11	−0.01 ± 0.33	0.524	1.000	0.396	0.672
Δ flattest K (D)	0.83 ± 5.82	−0.03 ± 0.40	0.04 ± 0.53	0.598	0.627	0.521	1.000
Δ steepest K (D)	0.89 ± 6.14	0.03 ± 0.52	−0.13 ± 0.62	0.324	0.873	0.540	0.205
Baseline and 12 mos							
Δ spherical equivalent (D)	−0.27 ± 0.72	−0.35 ± 0.59	−0.44 ± 1.02	**0.033**	0.279	**0.014**	0.281
Δ axial length (mm)	0.28 ± 0.27	0.21 ± 0.19	0.27 ± 0.28	0.299	0.206	1.000	0.396
Δ average K (D)	0.88 ± 5.96	0.02 ± 0.32	−0.06 ± 0.45	0.488	1.000	0.462	0.437
Δ flattest K (D)	0.81 ± 5.81	0.02 ± 0.56	−0.07 ± 0.51	0.248	1.000	0.201	0.248
Δ steepest K (D)	0.93 ± 6.12	0.02 ± 0.48	−0.05 ± 0.53	0.546	0.776	0.409	0.965

D = diopter; Δ = change; K = corneal curvature. The Overall P Value was obtained using the Kruskal–Wallis test, while the P Value (in Pair Comparisons) was derived from Dunn’s test for multiple comparisons. *P < 0.05 was considered statistically significant. The bold values means statistical significant.

As presented in Table 3, there were no significant differences in average corneal curvature, K1, or K2 values across the three treatment groups at baseline or throughout follow-up. Repeated measures analysis confirmed that there were no significant changes in keratometric parameters over time (all P > 0.05). These results suggest that low-concentration atropine does not influence anterior segment curvature during the treatment period.

**TABLE 3:** Corneal parameters at each time point.

​	0.05% atropine (n = 59)	0.025% atropine (n = 66)	0.01% atropine (n = 56)	Group overall P value	Time
Average K (D)	​	​	​	​	0.602
1 mos	42.91 ± 5.82	43.56 ± 1.44	43.55 ± 1.31	0.872	​
3 mos	43.59 ± 1.28	43.57 ± 1.29	43.57 ± 1.41	0.994	​
6 mos	43.65 ± 1.27	43.27 ± 2.83	43.59 ± 1.38	0.737	​
12 mos	43.66 ± 1.37	43.57 ± 1.37	43.53 ± 1.26	0.870	​
Flattest K (D)	​	​	​	​	0.005
1 mos	43.01 ± 1.25	42.95 ± 1.41	42.79 ± 1.26	0.512	​
3 mos	42.92 ± 1.24	42.94 ± 1.22	42.83 ± 1.33	0.889	​
6 mos	42.97 ± 1.24	42.93 ± 1.37	42.86 ± 1.34	0.891	​
12 mos	42.95 ± 1.32	42.98 ± 1.44	42.74 ± 1.24	0.593	​
Steepest K (D)	​	​	​	​	<0.001
1 mos	44.28 ± 1.37	44.16 ± 1.52	44.31 ± 1.46	0.844	​
3 mos	44.26 ± 1.41	44.20 ± 1.42	44.30 ± 1.55	0.928	​
6 mos	44.33 ± 1.39	44.16 ± 1.43	44.24 ± 1.56	0.824	​
12 mos	44.37 ± 1.50	44.15 ± 1.46	44.32 ± 1.39	0.701	​

D = diopters; K = corneal curvature. The overall group comparisons were assessed using ANOVA, for parametric data or Kruskal–Wallis tests for nonparametric distributions. Longitudinal treatment effects were then evaluated at each follow-up interval through Friedman tests analyzing paired ocular measurements. Normally distributed data: mean ± SD; Non-normally distributed data: median (IQR); *P < 0.05 was considered statistically significant.

Linear regression analyses identifying predictors of SE change are shown in Table 4. In all three treatment groups, axial length elongation was a significant predictor of SE progression (P < 0.01). The adjusted *R* ^2^ was highest in the 0.05% group (34.8%), followed by the 0.025% group (34.2%) and lowest in the 0.01% group (14.2%), indicating that the contribution of AL to refractive change varied by atropine concentration. After adjusting for age and sex (Model 2), axial length remained the only significant predictor across all models, while neither age nor gender contributed significantly to SE progression (all P > 0.05). Seemingly unrelated regression analysis confirmed that the regression coefficients of AL on SE were not statistically different across groups.

**TABLE 4:** Linear regression for change in spherical equivalent and ocular biometrics.

Variable	0.05% atropine			0.025% atropine			0.01% atropine		
	*β Coefficient*	*Standard error*	*P Value*	*β Coefficient*	*Standard error*	*P Value*	*β Coefficient*	*Standard error*	*P Value*
Model 1									
Δ axial length (mm)	−1.61	0.30	<0.001	−1.81	0.33	<0.001	−1.48	0.47	0.003
Δ adjusted R2 (%)	34.84	​	​	34.24	​	​	14.21	​	​
Model 2									
Δ axial length (mm)	−1.58	0.31	<0.001	−1.96	0.35	<0.001	−1.34	0.48	0.008
Gender (F0, M1)	−0.17	0.16	0.295	0.08	0.13	0.573	−0.02	0.27	0.931
Age	0.03	0.04	0.538	−0.05	0.04	0.266	0.12	0.09	0.183
Δ adjusted R2 (%)	34.01	​	​	34.30	​	​	14.20	​	​

D = diopter; Δ = change over 1 year. Models 1 is the equations using a stepwise selection procedure in linear regression models. Model 2 is the equation using an enter procedure in the linear regression model. P < 0.05 was considered statistically significant.

No serious adverse events related to atropine were reported during the study period. Photophobia was the most commonly reported side effect, occurring in 6 children in the 0.05% atropine group, 1 child in the 0.025% group, and 1 child in the 0.01% group. Allergic reactions were rare, with 4 cases of allergic conjunctivitis reported, 1 in the 0.025% group and 1 in the 0.01% group. Notably, no participants in any group reported near-vision blur.

## Discussion

This prospective, real-world, multi-center study evaluated and compared the efficacy and safety of 0.01%, 0.025%, and 0.05% atropine eye drops in slowing myopia progression among Chinese children. Consistent with previous randomized clinical trials, our findings demonstrated a dose-dependent effect, with 0.05% atropine showing the most reduction in SE progression, and 0.025% atropine exhibiting the least axial elongation. These results support the growing evidence base for low-concentration atropine as an effective pharmacological strategy in pediatric myopia control.

Previously, the landmark ATOM1 trial provided strong placebo-controlled evidence for the efficacy of 1% atropine, with a 77% reduction in SE progression over 2 years and virtually no axial elongation (−0.02 mm), while concerns regarding side effects and the rebound effect during the washout period (−1.14 D/year in the atropine group) tempered its clinical utility (Chia et al., 2016). To identify a more optimal concentration, the ATOM2 study compared 0.5%, 0.1%, and 0.01% atropine (Chia et al., 2012). While 0.01% atropine showed modest SE control (−0.49 D/2 years) and limited axial length suppression (0.41 mm), it had significantly fewer side effects and minimal rebound (−0.28 D) during washout, supporting its use as a safer alternative to high-dose regimens.

Nonetheless, the absence of a placebo group in ATOM2 prompted the initiation of the LAMP study (Yam et al., 2019; Huang et al., 2016), a randomized, double-blind, placebo-controlled trial that directly compared 0.01%, 0.025%, and 0.05% atropine. LAMP confirmed a clear concentration-dependent effect: after 1 year, SE progression was −0.27 D in the 0.05% group, −0.46 D in the 0.025% group, and −0.59 D in the 0.01% group, while axial elongation was 0.20 mm, 0.29 mm, and 0.36 mm, respectively. Notably, 0.05% atropine demonstrated the highest efficacy, and even 0.01% atropine resulted in a 12% reduction in AL growth and a 27% reduction in refractive error progression compared to placebo. Importantly, all concentrations were well tolerated with minimal adverse effects, supporting the clinical utility of low-dose atropine for myopia control.

Our findings are consistent with those of the LAMP trial. We observed a significantly lower SE progression in the 0.05% group compared to 0.01% atropine, and while AL elongation did not differ statistically among groups, the 0.025% group exhibited the lowest mean increase in axial length. Linear regression models showed that axial elongation was a strong predictor of SE progression, particularly in the 0.05% and 0.025% groups, which had the highest adjusted *R* ^2^ values (34.8% and 34.2%, respectively). These results suggest that both concentrations exert meaningful control over structural eye growth, which is critical for reducing the risk of myopia-related complications.

Additional evidence from meta-analyses and randomized trials has further supported the safety and efficacy of low-dose atropine. Huang et al. (2016) reported that atropine, across high, moderate, and low doses, outperformed placebo or single vision lenses, with high-dose atropine (1% and 0.5%) being most effective. Wang et al. (2017) also demonstrated that 0.5% atropine led to significantly less SE progression and axial elongation compared to placebo, without serious adverse events. Study by Tan et al. (2019) further explored combination therapies (e.g., atropine with orthokeratology), showing enhanced control of axial elongation.

In this study, although the 0.05% atropine group showed the statistically lowest SE progression, the 0.025% group exhibited the numerically lowest AL elongation. We prioritized AL elongation as the primary indicator for efficacy for several reasons. Primarily, axial elongation is the most objective structural biomarker for myopic progression, as SE can be influenced by transient factors such as accommodation. More importantly, the long-term goal of myopia control is to reduce the risk of vision-threatening complications which are directly governed by axial length rather than refractive error alone. Furthermore, considering the concentration-dependent side effects associated with atropine, such as photophobia and decreased near-point accommodation, the 0.025% concentration may offer a more favorable ‘sweet spot.’ It achieves axial control comparable to 0.05% while potentially reducing the burden of side effects, thereby improving long-term treatment adherence in pediatric populations.

Recently, Wei et al. (2020) conducted a randomized, placebo-controlled trial in China, finding that 0.01% atropine reduced myopia progression by 34.2% and axial elongation by 22.0% over 1 year. Similarly, the GTAM study in Spain (Perez-Flores et al., 2021) confirmed the efficacy and safety of 0.01% atropine in a real-world multicenter setting, reporting SE progression of −0.44 D and AL increase of 0.27 mm, with significantly fewer side effects and a notable reduction compared to the prior year’s progression.

This study provides essential Real-World Evidence (RWE) that complements the findings of previous landmark Randomized Controlled Trials (RCTs). While RCTs establish efficacy under ideal, highly controlled conditions, our RWE reflects the complexities of routine clinical practice. The “Real-World” nature of this cohort is characterized by the physician-led titration of atropine concentrations based on individual treatment response, rather than a fixed-dose protocol. Furthermore, our study population encompasses a wider spectrum of age and baseline refractive errors, providing results with high generalizability (external validity) to the general pediatric population. Taken together, our results contribute to the accumulating body of RWE supporting low-dose atropine use, particularly 0.025% and 0.05%, in East Asian pediatric populations. While 0.01% remains a safe and minimally invasive option, its clinical effect may be insufficient in children with rapid myopia progression. Conversely, although 0.05% offers stronger efficacy, tolerability may vary across individuals. Our study suggests that 0.025% atropine offers a favorable balance between efficacy and side effect profile, potentially serving as a well choice in routine clinical practice.

However, several limitations should be noted in this study. First, the study was not designed as a randomized controlled trial, group allocation was based on parental preference and physician recommendation, which may introduce selection bias and limit the generalizability of the findings. Second, although efforts were made to standardize follow-up and data collection, the potential for unmasking remains, particularly due to observable side effects such as photophobia and reduced accommodation, which may have led participants or caregivers to infer treatment allocation, as has been noted in previous atropine trials. Lastly, the follow-up period of 12 months may not be sufficient to fully assess long-term treatment effects and rebound risks following cessation. Therefore, future large-scale, randomized, double-masked, placebo-controlled studies with extended follow-up are needed to confirm the long-term efficacy, safety, and optimal concentration of low-dose atropine, particularly 0.025%, across diverse pediatric populations.

## Conclusion

In this prospective study of Chinese children with myopia, all three concentrations of low-dose atropine (0.01%, 0.025%, and 0.05%) demonstrated efficacy in slowing refractive progression, with a clear dose-dependent trend. Among them, 0.025% atropine provided a favorable balance between efficacy and safety, showing comparable control of axial elongation to 0.05% atropine, with potentially fewer side effects. Compared to 0.01%, it offered superior control of both spherical equivalent and axial length progression. These findings support 0.025% atropine as a clinically optimal concentration for routine myopia management in school-aged children. Further long-term, randomized studies are needed to validate its safety profile and guide individualized treatment strategies.

## Data Availability

The datasets used and/or analyzed during the current study are available from the corresponding authors on reasonable request.

## References

[B1] Chia A. Chua W. H. Cheung Y. B. Wong W. L. Lingham A. Fong A. (2012). Atropine for the treatment of childhood myopia: safety and efficacy of 0.5%, 0.1%, and 0.01% doses (atropine for the treatment of Myopia 2). Ophthalmology 119 (2), 347–354. 10.1016/j.ophtha.2011.07.031 21963266

[B2] Chia A. Lu Q. S. Tan D. (2016). Five-year clinical trial on atropine for the treatment of myopia 2: myopia control with atropine 0.01% eyedrops. Ophthalmology 123 (2), 391–399. 10.1016/j.ophtha.2015.07.004 26271839

[B3] Clark T. Y. Clark R. A. (2015). Atropine 0.01% eyedrops significantly reduce the progression of childhood myopia. J. Ocul. Pharmacol. Ther. 31 (9), 541–545. 10.1089/jop.2015.0043 26218150

[B4] Hieda O. Hiraoka T. Fujikado T. Ishiko S. Hasebe S. Torii H. (2021). Efficacy and safety of 0.01% atropine for prevention of childhood myopia in a 2-year randomized placebo-controlled study. Jpn. J. Ophthalmol. 65 (3), 315–325. 10.1007/s10384-021-00822-y 33586090

[B5] Holden B. A. Fricke T. R. Wilson D. A. Jong M. Naidoo K. S. Sankaridurg P. (2016). Global prevalence of myopia and high myopia and temporal trends from 2000 through 2050. Ophthalmology 123 (5), 1036–1042. 10.1016/j.ophtha.2016.01.006 26875007

[B6] Huang J. Wen D. Wang Q. McAlinden C. Flitcroft I. Chen H. (2016). Efficacy comparison of 16 interventions for myopia control in children: a network meta-analysis. Ophthalmology 123 (4), 697–708. 10.1016/j.ophtha.2015.11.010 26826749

[B7] Pan Z. Xian H. Li F. Wang Z. Li Z. Huang Y. (2025). Myopia and high myopia trends in Chinese children and adolescents over 25 years: a nationwide study with projections to 2050. Lancet Reg. Health West Pac 59, 101577. 10.1016/j.lanwpc.2025.101577 40568343 PMC12192684

[B8] Perez-Flores I. Macias-Murelaga B. Barrio-Barrio J. (2021). A multicenter Spanish study of atropine 0.01% in childhood myopia progression. Sci. Rep. 11 (1), 21748. 34741059 10.1038/s41598-021-00923-1 PMC8571279

[B9] Tan Q. Ng A. L. Cheng G. P. Woo V. C. Cho P. (2019). Combined atropine with orthokeratology for myopia control: study design and preliminary results. Curr. Eye Res. 44 (6), 671–678. 10.1080/02713683.2019.1568501 30632410

[B10] Wang Y. R. Bian H. L. Wang Q. (2017). Atropine 0.5% eyedrops for the treatment of children with low myopia: a randomized controlled trial. Med. Baltim. 96 (27), e7371. 10.1097/MD.0000000000007371 28682887 PMC5502160

[B11] Wei S. Li S. M. An W. Du J. Liang X. Sun Y. (2020). Safety and efficacy of low-dose atropine eyedrops for the treatment of myopia progression in Chinese children: a randomized clinical trial. JAMA Ophthalmol. 138 (11), 1178–1184. 10.1001/jamaophthalmol.2020.3820 33001210 PMC7530823

[B12] Wei J. Xiang X. Zhang P. Mu J. Lv H. Duan J. (2024). Large-scale study in chengdu, China: the prevalence of myopia full-correction decreased with increasing myopia in adolescents. Heliyon 10 (11), e31593. 10.1016/j.heliyon.2024.e31593 38841481 PMC11152689

[B13] Xu M. Zhang F. (2025). Advances in optical and pharmacological strategies for myopia correction in children. Am. J. Transl. Res. 17 (4), 2422–2433. 10.62347/GZUA2622 40385000 PMC12082519

[B14] Yam J. C. Jiang Y. Tang S. M. Law A. K. P. Chan J. J. Wong E. (2019). Low-concentration atropine for myopia progression (LAMP) study: a randomized, double-blinded, placebo-controlled trial of 0.05%, 0.025%, and 0.01% atropine eye drops in myopia control. Ophthalmology 126 (1), 113–124. 10.1016/j.ophtha.2018.05.029 30514630

